# Volatile Oil Chemical Composition of Wild, Edible *Centaurea scabiosa* L. and Its Cytotoxic Activity

**DOI:** 10.3390/plants11233267

**Published:** 2022-11-28

**Authors:** Ivana Carev, Anja Golemac, Sonja Siljak-Yakovlev, Francois Xavier Pellay, Olivera Politeo

**Affiliations:** 1 Department of Biochemistry, Faculty of Chemistry and Technology, University of Split, Ruđera Boškovića 35, 21000 Split, Croatia; 2 NAOS Institute of Life Science, 355, rue Pierre-Simon Laplace, 13290 Aix, France; 3 Mediterranean Institute for Life Science, Meštrovićevo šetalište 45, 21000 Split, Croatia; 4 Ecologie Systématique Evolution, CNRS, AgroParisTech, Université Paris-Saclay, 91190 Gif-sur-Yvette, France

**Keywords:** biological activity, *Centaurea scabiosa*, GC/MS, chromosome number, genome size, volatile oil

## Abstract

*Centaurea* species are well known as a source of phytopharmaceuticals having both beneficial and harmful influences on human health. *Centaurea scabiosa* L. is a wild edible plant used in Mediterranean cuisine in the Dalmatian region of Croatia. We have assessed the volatile oil’s chemical composition using GC/MS chromatography and its cytotoxic activity on human fibroblasts using the MTT test. Data on chromosome number, obtained by classical karyological methods, and genome size, assessed by flow cytometry, of the same plant material of *C. scabiosa*, were also given. The major chemical compounds found in *C. scabiosa* volatile oil were heptacosane, caryophyllene oxide, alloaromadendrene epoxide, α-cyperone, and *α*-bisabolol. This volatile oil showed no cytotoxicity on human fibroblasts in a dose range of 0.01–1 g/L. The chromosome number of a *C. scabiosa* sample from Croatia showed 2n = 20 + 2B chromosomes. The total genome DNA amount of 2C = 3.3 ± 0.01 pg or 1 Cx = 1628 Mbp presents the first report on the genome size of this species from Croatia. The presented results support the idea of using this plant in the human diet. To our knowledge, this is the first report on edible *C. scabiosa* species in general and in particular from Croatia.

## 1. Introduction

The *Centaurea* L. genus (Asteracea family) comprises around 500–600 annual, biannual, and perennial herbaceous plants distributed around the world but only north of the equator, mostly in the Eastern Hemisphere with the diversification center in the Middle East. Many of these species have been used in traditional medicine for the treatment of various diseases and conditions [1].

Plants and their extracts can be valuable sources of pharmacologically active compounds such as antibiotics, anti-cancer agents, and neuroactive molecules. *Centaurea* species are known as a source of phytopharmaceuticals that could have both beneficial and harmful influences on human health. Among the many *Centaurea* species already known for their medicinal properties, only a few have been known for their use in human diets [2,3]. 

Harvesting wild plants and using them for medicinal purposes and in the diet is part of traditional plant use in the coastal Mediterranean area. Due to high biodiversity and ethnopharmacology practice, the people of Croatia and the Dalmatian region, as well as the south Herzegovina region in Bosnia and Herzegovina, use an unusually large number of wild plant species for medicinal purposes and diet. *Centaurea scabiosa* is well known in the Eastern and Western Mediterranean for its pharmacological properties and is a wild edible plant used in the Mediterranean cuisine of the Dalmatian region (Croatia). It is used as a raw food, and its volatile compounds from volatile oil are present in the diet [4,5,6]. 

*Centaurea scabiosa* has also been used for medical purposes, such as the treatment of scabies and other skin complaints, which is where the root of its scientific name, *scabiosa*, comes from. It is known that the roots and seeds of *C. scabiosa* are used in traditional medicine for wound healing, to treat kidney problems and mouth ulcers, as well as for tonic and diuretic purposes [7].

*Centaurea scabiosa* non-volatile extracts, studied so far, were assessed for the presence of phenolic components, flavonoids, and some sesquiterpene lactones. They were tested for antimicrobial and antioxidant activity [8,9]. 

The *Centaurea* genus is very difficult taxonomically due to its large morphological, karyological, and palynological diversity. Apart from the detailed morphological study, genome size and chromosome number assessments present additional approaches that assist in the identification of the studied plant material [10,11,12,13]. 

The aim of our study was to:

(a) Assess the volatile oil chemical composition of *C. scabiosa* using the GC/MS technique;
(b) Test *C. scabiosa* volatile oil cytotoxicity using the MTT assay on human fibroblasts at a concentration dose range of 0.01–1 g/L;
(c) Determine genome size using flow cytometry and chromosome number using the classical karyological method.

Depending on the results obtained, this species could be proposed for use in human food.

## 2. Results and Discussion

The chemical composition of *C. scabiosa* hydro-distilled volatile oil was determined using the GC/MS chromatographic technique and is shown in Table 1.

The 32 detected volatile oil chemical compounds represent 90.21% of volatile oils. The chemical compounds are grouped in terpenes (43.73%), consisting of non-oxygenated sesquiterpenes (1.23%), oxygenated sesquiterpenes (41.09%), and diterpenes (1.41%); nonterpene compounds consisting of hydrocarbons (31.10%), aldehydes (3.67%), acids (7.00%), esters (4.03%), and other compounds (0.68%). The dominant chemical compounds in the studied volatile oils were heptacosane (19.62%), caryophyllene oxide (10.90%), alloaromadendrene epoxide (10.27%), α-cyperone (8.16%), and *α*-bisabolol (4.99%) (Table 1). 

The common constituents of *Centaurea* species belong to a group of terpenes called sesquiterpenes, and they are usually dominant components in most of the *Centaurea* volatile oils [14,15,16,17,18,19,20,21,22,23,24,25]. Along with the sesquiterpenes, non-oxygenated hydrocarbon derivatives are dominantly present in most of the *Centaurea* species’ volatile oils, while oxygenated hydrocarbon derivatives are present in a smaller amount than non-oxygenated derivatives [14,15,16,17,18]. 

On the contrary, the studied *C. scabiosa* volatile oil demonstrated dominant compounds belonging to a group of oxygenated sesquiterpenes, while the non-oxygenated sesquiterpenes were present in a very small amount of 1.23% in total. While caryophyllene oxide can be found among the dominated compounds in *Centaurea* volatile oil, the presence of alloaromadendrene epoxide is rather exceptional, especially among dominant components, and this is the first report of this chemical compound as a dominant component of *Centaurea* volatile oil. It is also rare to find *α*-cyperone as a dominant compound in *Centaurea* volatile oils, as well as *α*-bisabolol [26]. Monoterpenes are usually present in amounts less than 1% in *Centaurea* species, and in the studied volatile oil of *C. scabiosa*, the monoterpenes were not present at all. Heptacosane was among the dominant components in *C. scabiosa*, which can also be found in some other *Centaurea* volatile oils [14,15,16,17,18,19,20,21,22,23,24,25]. 

It is interesting to point out that the name of the species *scabiosa* is derived from the skin disease scabies, and *α*-bisabolol, which is found in studied essential oils, is known for its effects on skin repair and wound healing [27].

The common constituents of *Centaurea* volatile oils, such as germacrene D and spathulenol, in our studied volatile oil were found in less than 1%. This is unusual for most of the *Centaurea* species, as these sesquiterpenes are commonly present as dominant constituents of *Centaurea* volatile oils. Hexadecanoic acid, another very common constituent of *Centaurea* volatile oils, was found in a small amount in the studied volatile oil [14,15,16,17,18,21,22,23].

The volatile oil of *C. scabiosa* studied revealed some distinct characteristics in the chemical composition of dominant components. It showed diversity in chemical composition compared to most of the *Centaurea* species, while showing some similarities with the chemical composition of volatile oils from the *Lopholoma* section, where *C. scabiosa* belongs [14,16,17,18,24]. Nonetheless, the dominant component combination is unique and first reported for this species, as is the presence of alloaromadendrene epoxide and α-cyperone as dominant components in *Centaurea* volatile oil.

**Figure 1:**
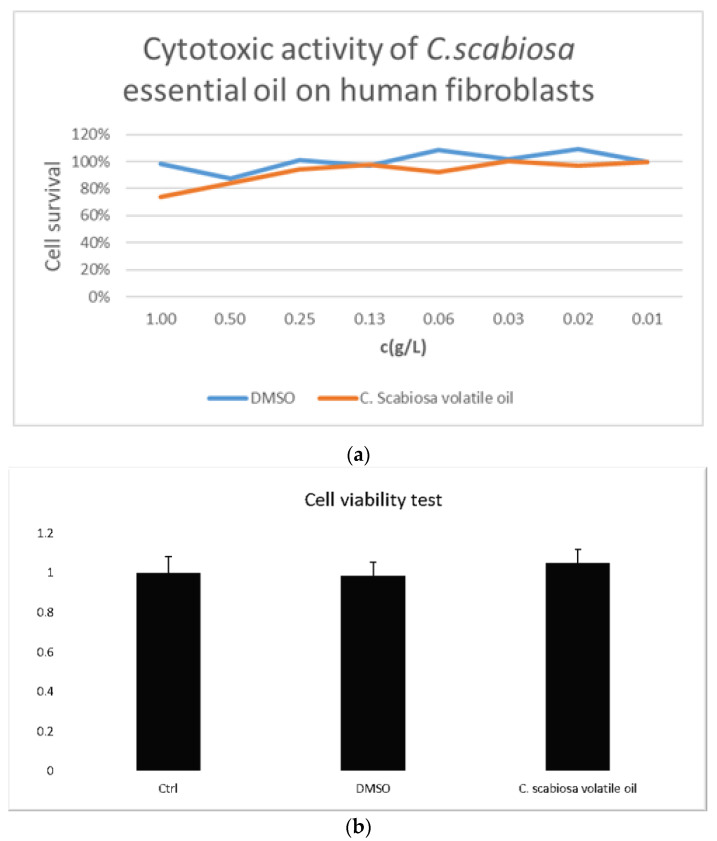
*Centaurea scabiosa* volatile oil tested on primary human skin fibroblasts with the MTT assay in eight different concentrations in the range of 0.01–1 g/L (**a**) The result for essential oil presents the average of the results for eight different concentrations of *C. scabiosa* essential oils, in triplicates (**b**).

*Centaurea scabiosa* volatile oil tested on primary human skin fibroblasts with the MTT assay showed no toxicity in a range of concentrations from 0.01 to 1 g/L with DMSO and water as controls (Figure 1). *Centaurea scabiosa* volatile oil tested on primary human skin fibroblasts with the MTT assay at five concentrations between 0.01 and 1 g/L showed there was no statistical difference from the control conditions, either water or DMSO, and therefore there is no toxicity. This has been supported by a test with propidium iodide that confirmed MTT results on the lack of toxicity of both tested extracts. 

We compared the biological activity of the studied volatile oils to known volatile oils of edible *Centaurea* species because *Centaurea* species are very poorly known phytochemically and phytopharmacologically, on their chemical composition of volatiles or non-volatiles, and even less on their biological activity. 

In the literature, some of the *Centaurea* species from Turkey and Italy have been mentioned as edible plants: *Centaurea cheiranthifolia* Willd. *Var. cheiranthifolia*; *C. cheiranthifolia* Willd. *Var. purpurascens* (DC.) Wagenitz, *C. cyanus* L., *C. depressa* Bieb., *C. glastifolia* L., *C. iberica* Trev. Ex Sprengel, *C. solstitialis* L. subsp. *solstitialis*, *C. haradjianii*, *C. jacea*, and *C. calcitrapa*. These species are mostly used in cooked or fresh salads, but no in-depth chemical analysis of the volatile oils of the mentioned species has been done in the cited literature [28,29]. 

Because there is a lack of data on the cytotoxic activity of the essential oils of the edible *Centaurea* plants mentioned, we used available literature data on the chemical composition of *C. cyanus* L., *C. depressa* Bieb., *C. iberica* Trev. Ex Sprengel, *C. solstitialis* L. *subsp*. *solstitialis*, *C. jacea*, and *C. calcitrapa* for discussion [21,22,30,31,32,33,34,35,36].

*Centaurea cyanus* L. and *C. depressa* Bieb., among their dominant components, had hexadecenoic acid, dodecanoic acid, and carvacrol, while *C. depressa* had tetradecanoic acid as well [26]. *C. depressa* was found to contain piperitone, elemol, β-eudesmol, and spathulenol as dominant components in another study [30]. The dominant components of *C. iberica* were arachidic acid, hexadecanoic acid, choleic acid, and isononane, while another record reported cyclosativene, dodecanoic acid, hexadecanoic acid, and tricosane as dominant components [31,32]. *Centaurea jacea* had two reports: one declared caryophyllene oxide, spathulenol, hexadecenoic acid, and 9-octadecanoic acid as dominant components, while the other listed germacrene D, hexahydrofarnesyl acetone, and ledol [21,33]. *Centaurea solstitialis* was the most studied, with four records on essential oil chemical composition, including bornyl acetate, limonene, and β-selinene in the first, hexadecenoic acid, heptacosane, and nonacosane in the second, and n-heneicosane, hexadecanoic acid, n-tricosane, n-pentacosane, and caryophyllene oxide in the third, and fourthly hexadecanoic acid, α-linolenic acid, germacrene D, and heptacosane [22,34,35,36]. As we can see, the dominant components of the essential oils from wild edible *Centaurea* species vary greatly, and only a few of the listed components overlap with those in *C. scabiosa*. 

Previously reported essential oils that were tested for cytotoxic activity using the MTT test were isolated from *C. cineraria*, *C. cyanus*, *C. behen*, *C. hajastana*, and *C. irritans*. All of the mentioned essential oils showed cytotoxic activity on different cell lines: *C. cineraria*, with cyclosativene and tetracyclic sesquiterpene as dominant components in essential oil, was tested on neurons and neuroblastoma; *C. cyanus* with hexadecanoic and linoleic acid as dominant components in essential oil, was tested on the HT29 cell line; *C. behen* was tested on human blood cultures, but the essential oil and composition was not reported; *C. hajastana* with β eudesmol, β caryophyllene, germacrene D, and caryophyllene oxide as dominant components in essential oil were tested on human liver cancer cells (HepG2); and *C. irritans* with oxygenated monoterpenes as dominant components in essential oil was tested on breast lung cells (MCF 7). Tested essential oils showed high variability in their chemical composition. Comparing the studied essential oil with those previously studied and tested for cytotoxicity, we have found similar dominant components only with *C. hajastana* for caryophyllene oxide. [3,37,38,39,40]. 

The classical karyological method revealed 2n = 20 + 2B chromosomes in *C. scabiosa*. Genome size, assessed with flow cytometry, was 2C = 3.3 ± 0.01 pg or 1 Cx = 1628 Mbp and presents the first data on the DNA amount of this species from Croatia. The basic chromosome number in the *Centaurea* genus ranges from x = 7 to 16, and several ploidy levels, mainly diploid (2x) and tetraploid (4x), are presented. Previous data reported on the genome size of this species were 2.60 pg, 3.58 pg, and 3.54 pg [41,42]. Our data on chromosome number and genome size were in compliance with the previous data for diploid samples of this species and present data for easier authentication of plant material used for this phytochemical study [10,12]. 

Our study contributes to the body of knowledge on this plant genera by filling a gap in scientific data on phytochemistry and phytopharmacology. The presented data on the volatile oil chemical composition of *C. scabiosa*, a wild edible plant used in the Mediterranean human diet, may serve as a list of potentially bioactive chemical compounds presented as major and minor non-nutrient components of food. The absence of toxicity in human fibroblasts supports the idea of using this plant in the human diet. 

## 3. Materials and Methods

### 3.1. Plant Material 

*Centaurea scabiosa* L. plant material (leaves, stems, and flowers) was collected in July 2016 from wild growing populations in the coastal area of Croatia, in a village named Tijarica (X = 5,652,083; Y = 4,830,593), at an elevation of 645 m, growing on a dark brown soil type. The authentication of plant material was carried out by means of macroscopic traits. *Centaurea scabiosa* was additionally characterized by genome size estimation using flow cytometry and by chromosome number. Voucher specimens (2016_Cscabiosa_25_014) of plant materials used for this study have been deposited, with the date and location of collection, in the herbarium at the Department of Biochemistry, Faculty of Chemistry and Technology, Split, Croatia. On 4 May 2021, the plant name was checked with http://www.theplantlist.org for the last time. The leaves, stems, and a few flowers were used for aqueous and volatile oil extraction, while germinated seeds from the same individual plants were used for cytogenetic assessment and plant authentication.

### 3.2. Volatile Oil Extraction, Gas Chromatography (GC), and Gas Chromatography—Mass Spectrometry (GC–MS) Analyses

The volatile oil from extracted air-dried aerial parts of *C. scabiosa* was hydro-distilled using Clavenger apparatus for 3 h and stored in a sealed vial, under −20 °C until use. The gas chromatography analysis of EO was performed using a Varian Inc. gas chromatograph, model 3900, Lake Forest, CA, USA. The gas chromatograph was equipped with a flame ionization detector and mass detector, model 2100T, and a non-polar capillary column, VF-5MS (30 m × 0.25 mm i.d.; coating thickness 0.25 mm). The temperature program for the VF-5MS column was: 60 °C isothermal for 3 min, then increased to 246 °C at a rate of 3 °C min^−1^ and held isothermal for 25 min. The carrier gas was helium at a flow rate of 1 mL min^−1^, injector temperature was 250 °C, injected volume was 1 μL, split ratio was 1:20, and the FID detector temperature was 300 °C. Mass spectrometer ionization voltage was 70 eV, the mass scan range was 40–350 mass units, and the ion-source temperature was 200 °C. The percentages of components were calculated mathematically as mean values from the GC and GC-MS peak areas. Identification of EO chemical composition was based on comparison of compound mass spectra with databases (Wiley 7 library—Wiley, New York, NY, USA) and comparison of their retention indices, relative to a series of n-alkanes C_9_–C_40_, with an internal database created during previous analyses, and literature retention indices using NIST 2002 (National Institute of Standards and Technology, Gaithersburg, MD, USA) [43]. 

### 3.3. Toxicity on Human Primary Fibroblasts

The toxicity of *C. scabiosa* volatile oil was measured in primary fibroblasts through the MTT assay. To prepare a stock solution, essential oil was dissolved in dimethyl sulfoxide (DMSO) prior to cell treatment. Furthermore, when preparing working solutions for cell application, stock solutions were dissolved in cell culture growth medium. The concentrations used for biological activity testing were obtained through serial dilution in the 0.01–1 g/L range. Medium RPMI-1640 and Dulbecco’s phosphate-buffered saline were obtained from Sigma-Aldrich. Bovine serum, L-glutamine, the penicillin-streptomycin antibiotic, and trypsin-EDTA were obtained from Gibco by Life Technologies. Human skin primary fibroblasts were purchased from Axol Bioscience Ltd., GB. Cells were incubated with RMPI supplemented with 1% penicillin-streptomycin, 1% L-glutamine, and 10% fetal bovine serum in a 37 °C, humidified, 5% CO_2_ incubator. Fibroblasts were seeded on a 96-well plate for testing and incubated with *Centaurea scabiosa* volatile oil (VO) extract for 24 h (when tested on cells). After 24 h of incubation with plant extracts, the medium was removed and replaced with a fresh one, and MTT working solution of 5 mg/mL of Thiazolyl Blue-Tetrazolium Bromide was added to medium. Cells were incubated for 4 h at 37 °C in a 5% CO_2_ incubator. After incubation, the medium was removed, and samples of essential oil in a range of 8 serial dilutions in the range of 0.01–1 g/L, dissolved in DMSO (Sigma Aldrich, Co., St. Louis, MO, USA), as well as DMSO controls without essential oil in related concentrations, were added. The absorbance of samples in biological and technical triplicates of each sample was read at 595 nm with the EnSight multimode plate reader (PerkinElmer, Waltham, MA, USA). Since all the concentrations tested did not show a toxic effect, the final presentation of the result was made as the average of all the results for the technical triplicates for all concentrations used.

### 3.4. Chromosome Number and Genome Size Evaluation 

Chromosome number was determined using the classical Feulgen technique from germinated seedlings of *C. scabiosa*. The cotyledons or first leaves were used for genome size assessment by flow cytometry, and *Solanum lycopersicum* L. ‘Montfavet 63–5′ (2C = 1.99 pg) was used as an internal standard [44]. Leaf samples and the internal standard were chopped together using a razor blade in a Petri dish with 600 μL of cold Gif Nuclear Isolation Buffer—GNB: 45 mM MgCl_2_, 30 mM sodium citrate, 60 mM MOPS (4-morpholine propane sulphonate, pH = 7), and 1% (*w*/*v*) polyvinylpyrrolidone 10,000, pH 7.2), containing 0.1% (*w*/*v*) Triton X–100, supplemented with 5 mM sodium metabisulphite and RNase (2.5 U/mL) [45]. The nuclei suspension was filtered through a nylon mesh (pore size 50 μm), to remove non-useful tissue fragments. The nuclei were stained with 50 μg/mL propidium iodide (Sigma Chemical Co., St. Louis, MO, USA) and flow cytometry was performed on a CytoFLEX S (Beckman Coulter- Life Science United States) with excitation at 561 nm, 30 mW; emission through a 610/20 nm band-pass filter. At least 5 individuals (biological replicates), measured in technical duplicates, with 5000–10,000 nuclei, were analyzed, and the average 2C DNA value was calculated using the linear relationship between the fluorescent signals from stained nuclei of known internal standards and the fluorescent signals from stained nuclei of the tested specimen.

### 3.5. Statistical Analysis

All statistical analyses were performed using the free software environment for statistical computing, the Microsoft Office package. 

## 4. Conclusions

This study presents a multidisciplinary approach to volatile oil chemistry and some biological traits of *C. scabiosa*, a wild edible species used in the Mediterranean diet. This is the first study of *C. scabiosa* volatile oil’s chemical composition and biological activity, toxicity on human fibroblasts, as well as the first assessment of this species’ genome size in Croatia. The novelty in the chemical composition of the volatile oil was the dominance of oxygenated sesquiterpenes, which is unusual for *Centaurea* species. The presence of alloaromadendrene epoxide and *α*-cyperone as dominant chemical compounds in the volatile oil of *C. scabiosa* was first reported for the *Centaurea* genus. The presence of α-bisabolol as a dominant compound was also rather rare and first reported in this amount, as well as the presence of heptacosane, which had never been reported in such a high concentration. The cytotoxic activity of volatile oil on human fibroblasts was found to be non-existent. The presented results support the idea of using *C. scabiosa* species in the human diet, indicating their safety for dietary consumption. 

## Figures and Tables

**Table 1:** Chemical composition and chemical class distribution of the essential oil of *Centaurea scabiosa* L.

	Compound Name		KI	Identification
	Terpene Compounds			
	*Non-oxygenated sesquiterpenes*	*1.23*		
1	Longifolene	0.43	1409	KI, MS
2	Aromadendrene	0.08	1421	KI, MS
3	*γ*-elemene	tr	1435	KI, MS
4	*cis*-*ß*-farnesene	tr	1443	KI, MS
5	*α*-hummulene	0.18	1452	KI, MS
6	*trans*-*ß*-farnesene	tr	1478	KI, MS
7	Germacrene D	0.54	1482	KI, MS
	*Oxygenated sesquiterpenes*	*41.09*		
8	Spathulenol	0.75	1568	KI, MS
9	Caryophyllene oxide	10.90	1583	KI, MS
10	Aromadendrene oxide	2.20	1626	KI, MS
11	Isospathulenol	3.52	1639	KI, MS
12	Alloaromadendrene epoxide	10.57	1655	KI, MS
13	*α*-bisabolol	4.99	1692	KI, MS
14	*α*-cyperone	8.16	1752	KI, MS
	*Oxygenated diterpene*	*1.41*		
15	Phytol	1.41	2119	KI, MS
	Non-terpene compounds			
	*Hydrocarbons*	*31.10*		
16	Tricosane	0.39	2300	KI, MS
17	Tetracosane	0.21	2400	KI, MS
18	Pentacosane	4.64	2500	KI, MS
19	Hexacosane	0.94	2600	KI, MS
20	Heptacosane	19.62	2700	KI, MS
21	Octacosane	0.52	2800	KI, MS
22	Nonacosane	4.78	2900	KI, MS
	*Aldehydes*	*3.67*		
23	Benzene acetaldehyde	0.35	1051	KI, MS
24	Longifolene aldehyde	3.32	1609	MS
	*Acids*	*7.00*		
25	Hexadecanoic acid	4.03	1977	KI, MS
26	*α*-linolenic acid	2.86	2165	KI, MS
27	Octadecanoic acid	0.11	2197	KI, MS
	*Esters*	*4.03*		
28	Benzoic acid methyl ester	3.64	1091	KI, MS
29	3,5-heptadienal-2-ethylidiene-6-methyl	0.39	1345	KI, MS
30	*Other compounds*	*0.68*		
31	4-vinylguaiacol	0.46	1330	KI, MS
32	Eugenol	0.22	1363	KI, MS

KI = Kovats retention index determined on a VF-5 MS column using the homologous series of *n*-alkanes (C_9_–C_40_); tr = traces (<0.1%); MS = mass spectra.

## Data Availability

Not applicable.

## References

[1] Abad M.J., Bedoya L.M., Bermejo P. (2013). Chapter 14—Essential Oils from the Asteraceae Family Active against Multidrug-Resistant Bacteria A2—Kon, Mahendra Kumar RaiKateryna Volodymyrivna. Fighting Multidrug Resistance with Herbal Extracts, Essential Oils and Their Components.

[2] Cakilcioglu U., Turkoglu I. (2010). An ethnobotanical survey of medicinal plants in Sivrice (Elazi{dotless}ĝ-Turkey). J. Ethnopharmacol..

[3] Manukyan A., Lumlerdkij N., Heinrich M. (2019). Caucasian endemic medicinal and nutraceutical plants: In-vitro antioxidant and cytotoxic activities and bioactive compounds. J. Pharm. Pharmacol..

[4] Łuczaj Ł., Zovko Končić M., Miličević T., Dolina K., Pandža M. (2013). Wild vegetable mixes sold in the markets of Dalmatia (southern Croatia). J. Ethnobiol. Ethnomed..

[5] Łuczaj Ł., Pieroni A. (2016). Nutritional ethnobotany in Europe: From emergency foods to healthy folk cuisines and contemporary foraging trends. Mediterranean Wild Edible Plants: Ethnobotany and Food Composition Tables.

[6] María de Cortes Sánchez-Mata J.T. (2016). Mediterranean Wild Edible Plants.

[7] Kenny O., Smyth T.J., Walsh D., Kelleher C.T., Hewage C.M., Brunton N.P. (2014). Investigating the potential of under-utilised plants from the Asteraceae family as a source of natural antimicrobial and antioxidant extracts. Food Chem..

[8] Sharonova N., Nikitin E., Terenzhev D., Lyubina A., Amerhanova S., Bushmeleva K., Rakhmaeva A., Fitsev I., Sinyashin K. (2021). Comparative assessment of the phytochemical composition and biological activity of extracts of flowering plants of *Centaurea cyanus* L., *Centaurea jacea* L., and *Centaurea scabiosa* L.. Plants.

[9] Kaminskiy I.P., Yermilova Y.V., Kadyrova T.V., Lar’Kina M.S., D’Yakonov A.A., Belousov M.V. (2019). Antiradical AC-tivity of extracts from siberian flora genus centaurea plants. Khimiya Rastit Syr’ya.

[10] Vallès J., Canela M.Á., Garcia S., Hidalgo O., Pellicer J., Sánchez-Jiménez I., Siljak-Yakovlev S., Vitales D., Garnatje T. (2013). Genome size variation and evolution in the family Asteraceae. Caryologia.

[11] Siljak-Yakovlev S., Solic M.E., Catrice O., Brown S.C., Papes D. (2005). Nuclear DNA content and chromosome number in some diploid and tetraploid Centaurea (Asteraceae: Cardueae) from the Dalmatia region. Plant Biol..

[12] Siljak-Yakovlev S., Pustahija F., Šolić E.M., Bogunić F., Muratović E., Bašić N., Catrice O., Brown S.C. (2010). Towards a Genome Size and Chromosome Number Database of Balkan Flora: C-Values in 343 Taxa with Novel Values for 242. Adv. Sci. Lett..

[13] Bennett M.D., Leitch I.J. (2011). Nuclear DNA amounts in angiosperms: Targets, trends and tomorrow. Ann. Bot..

[14] Flamini G., Tebano M., Cioni P.L., Bagci Y., Dural H., Ertugrul K., Uysal T., Savran A. (2006). A multivariate statistical approach to Centaurea classification using essential oil composition data of some species from Turkey. Plant Syst. Evol..

[15] Kilic O., Bagci E. (2016). Chemical Composition of Two Endemic *Centaurea* L. Taxa from Turkey, A Chemotaxonomic Approach. J. Essent. Oil-Bear. Plants.

[16] Zengin G., Aktumsek A., Boga M., Ceylan R., Uysal S. (2016). Essential Oil Composition of an Uninvestigated Centaurea Species from Turkey: Centaurea patula DC. J. Essent. Oil-Bear. Plants.

[17] Polatoglu K., Sen A., Bulut G., Bitis L., Goren N. (2014). Essential Oil Composition of Centaurea stenolepis Kerner. from Turkey. J. Essent. Oil Bear. Plants.

[18] Flamini G., Ertugrul K., Cioni P.L., Morelli I., Dural H., Bagci Y.G. (2002). E, Flamini K.; Cioni P.L.; Morelli I.; Dural H.; Bagci Y.G.E. Volatile constituents of two endemic Centaureaspecies from Turkey: *C. pseudoscabiosa* subsp. *pseudoscabiosa* and *C. hadimensis*. Biochem. Syst. Ecol..

[19] Novaković J., Rajčević N., Milanovici S., Marin P.D., Janaćković P. (2016). Essential Oil Composition of Centaurea atropurpurea and Centaurea orientalis Inflorescences from the Central Balkans—Ecological Significance and Taxonomic Implications. Chem Biodivers..

[20] Bruno M., Modica A., Catinella G., Canlı C., Arasoglu T., Çelik S. (2019). Chemical composition of the essential oils of *Centaurea tomentella* Hand.-Mazz. and *C. haussknechtii* Boiss. (Asteraceae) collected wild in Turkey and their activity on microorganisms affecting historical art craft. Nat. Prod. Res..

[21] Politeo O., Carev I., Veljaca A. (2019). Phytochemical Composition, Antiradical and Anticholinesterase Potentials of Centaurea alba and Centaurea jacea Volatile Oils. Croat. Chem. Acta.

[22] Carev I., Ruščić M., Skočibušić M., Maravić A., Siljak-Yakovlev S., Politeo O. (2017). Phytochemical and Cytogenetic Characterization of *Centaurea solstitialis* L. (Asteraceae) from Croatia. Chem. Biodivers..

[23] Carev I., Maravić A., Bektašević M., Ruščić M., Siljak-Yakovlev S., Politeo O. (2018). *Centaurea rupestris* L.: Cytogenetics, essential oil chemistry and biological activity. Croat. Chem. Acta.

[24] Riccobono L., Maggio A., Bruno M., Bancheva S., Santucci O., Senatore F. (2017). Chemical composition of the essential oil of Centaurea grinensis Reuter and Centaurea apiculata Ledeb: Growing wild in Croatia and Bulgaria, respectively and PCA analysis of subgenus Lopholoma (Cass.) Dobrocz. Plant Biosyst..

[25] Polatoğlu K., Şen A., Bulut G., Bitiş L., Gören N. (2014). Essential Oil Composition of Centaurea kilaea Boiss. and *C. cuneifolia* Sm. from Turkey. Nat. Vol. Essent. Oils.

[26] Karamenderes C., Demirci B., Baser K.H.C. (2008). Composition of essential oils of ten *Centaurea* L. taxa from Turkey. J. Essent. Oil Res..

[27] El-Lakany S.A., Abd-Elhamid A.I., Kamoun E.A., El-Fakharany E.M., Samy W.M., Elgindy N.A. (2019). α-Bisabolol-loaded cross-linked zein nanofibrous 3D-scaffolds for accelerating wound healing and tissue regeneration in rats. Int. J. Nanomed..

[28] Geraci A., Amato F., Di Noto G., Bazan G., Schicchi R. (2018). The wild taxa utilized as vegetables in Sicily (Italy): A traditional component of the Mediterranean diet. J. Ethnobiol. Ethnomed..

[29] Şenkardeş İ., Bulut G., Doğan A., Tuzlaci E. (2019). An ethnobotanical analysis on wild edible plants of the turkish asteraceae taxa. Agric. Conspec. Sci..

[30] Esmaeili A., Rustaiyan A., Nadimi M., Masoudi S., Tadayon F., Sedaghat S., Ebrahimpur N., Hajyzadeh E. (2005). Volatile Constituents of Centaurea depressa M.B. and *Carduus pycnocephalus* L. Two Compositae Herbs Growing Wild in Iran. J. Essent. Oil Res..

[31] Ertas A., Goren A.C., Boga M., Demirci S., Kolak U. (2014). Chemical Composition of The Essential Oils of Three Centaurea Species Growing Wild in Anatolia and Their Anticholinesterase Activities. J. Essent. Oil Bear. Plants.

[32] Erel S.B., Demir S., Nalbantsoy A., Ballar P., Khan S., Yavasoglu N.U., Karaalp C. (2014). Bioactivity screening of five Centaurea species and in vivo anti-inflammatory activity of C. athoa. Pharm. Biol..

[33] Milosevic T., Argyropoulou C., Solujic S., Murat-Spahic D., Skaltsa H. (2010). Chemical composition and antimicrobial activity of essential oils from Centaurea pannonica and C. jacea. Nat. Prod. Commun..

[34] Lograda M., Chalard P., Figueredo G., Khalfoune K., Silin H., Ramdani T. (2013). Phytochemistry, antibacterial activity and chromosome number of *Centaurea solstitialis* L. Grown in Algeria. Glob. J. Res. Med. Plants Indig. Med..

[35] Esmaeili A., Akbari M.T., Moazami N., Masoudi S., Amiri H.R.A. (2006). Composition of the Essential Oils of *Xanthium strumarium* L. and *Cetaurea solstitialis* L. from Iran. J. Essent. Oil Res..

[36] Senatore F., Formisano C., Raio A., Bellone G., Bruno M. (2008). Volatile components from flower-heads of *Centaurea nicaeensis* All., C-parlatoris Helder and *C-solstitialis* L. ssp schouwii (DC.) Dostal growing wild in southern Italy and their biological activity. Nat. Prod. Res..

[37] Ayromlou A., Masoudi S., Mirzaie A. (2020). Chemical composition, antioxidant, antibacterial, and anticancer activities of scorzonera calyculata boiss. And centaurea irritans wagenitz. Extracts, endemic to iran. J. Rep. Pharm. Sci..

[38] Çelikezen F.Ç., Hayta Ş., Özdemir Ö., Türkez H. (2019). Cytotoxic and antioxidant properties of essential oil of *Centaurea behen* L. in vitro. Cytotechnology.

[39] Mirzaie A., Karizi S.Z. (2016). Study of chemical composition and characteristics of Centurea cyanus extract on colon cancer cell line and analysis of apoptosis gene expression. Tehran. Univ. Med. J..

[40] Toğar B., Türkez H., Geyikoğlu F., Hacimüftüoğlu A., Tatar A. (2014). Antiproliferative, genotoxic and oxidant activities of cyclosativene in rat neuron and neuroblastoma cell lines. Arch. Biol. Sci..

[41] Bancheva S., Greilhuber J. (2006). Genome size in Bulgarian *Centaurea* S.L. (Asteraceae). Plant Syst. Evol..

[42] Munzbergova Z. (2009). The effect of genome size on detailed species traits within closely related species of the same habitat. Bot. J. Linn. Soc..

[43] Adams R.P. (2005). Identification of Essential Oil Components by Gas Chromatography/Mass Spectrometry.

[44] Lepers-Andrzejewski S., Siljak-Yakovlev S., Brown S.C., Wong M., Dron M. (2011). Diversity and dynamics of plant genome size: An example of polysomaty from a cytogenetic study of Tahitian vanilla (Vanilla× tahitensis, Orchidaceae). Am. J. Bot..

[45] Bourge M., Brown S.C., Siljak-Yakovlev S. (2018). Flow cytometry as tool in plant sciences, with emphasis on genome size and ploidy level assessment. Genet. Appl..

